# Equality over intentionality: The normative social preferences of neutral third-parties

**DOI:** 10.1371/journal.pone.0205240

**Published:** 2018-11-14

**Authors:** Ciril Bosch-Rosa

**Affiliations:** 1 Chair of Macroeconomics, Department of Economics, Berlin University of Technology, Berlin, Germany; 2 Department of Management and Business, Colegio Universitario de Estudios Financieros, Madrid, Spain; Universidad Loyola Andalucia, SPAIN

## Abstract

This paper studies whether intentionality is more prevalent than fairness in social preferences. We do this by introducing a new three-player game in which the choices of neutral third-party arbiters are isolated from any monetary or strategic concerns. This allows us to study the normative preferences of subjects, and to compare the relative weight they give to intentions and inequality. The results show that arbiters are mainly concerned with inequality, while other’s (selfish) intentions seem to play a minor role in their preferences. This result is robust to a series of experimental designs, suggesting that the role of intentions in social preferences might be smaller than implied by the previous literature.

## Introduction

“How selfish soever man may be supposed, there are evidently some principles in his nature, which interest him in the fortune of others, and render their happiness necessary to him, though he derives nothing from it except the pleasure of seeing it.”

*The Theory of Moral Sentiments*, Adam Smith (1759)

Social preferences are relevant in many research fields, from economics, philosophy [1] and conflict resolution [2], to anthropology [3] and evolutionary biology [4]. Some authors even consider social preferences to be one of the main pillars of legal studies [5]. A better understanding of social preferences is therefore not only relevant to refine models of fairness and cooperation, but can help understand many other fundamental questions about human behavior.

Yet, surprisingly, while it has been well established that both fairness and intentions are important factors in social preferences [6], it is not clear whether one of the two principles dominates. On the one hand [6–8] claim that intentions are the main driver behind the behavior of third (and second) party decision-makers, on the other [9] defends that intentions only matter in the negative domain of reciprocity, while [10–12] show that subjects respond more to outcomes than to intentions.

In this paper we contribute to this literature by introducing a three-player ultimatum game in which a proposer makes an offer on how to split $10 with a receiver (who plays no active role in the game), while a decision-maker fills in a strategy profile accepting or rejecting every potential offer the proposer can make. If the offer is accepted by the decision-maker, then the split takes place as suggested; if rejected, then both proposer and receiver get $0. The decision-maker is paid a “flat fee” independent of the outcome of the game.

This new design has several advantages over previous ones: First, we introduce a third-party with no strategic or monetary skin in the game, allowing us to study the “true” distributional preferences of decision-makers. This takes care of the concern raised by [13] that in most experimental setups the self-interest of decision-makers can obscure the measures of social preferences, allowing us to observe what [7] call “truly normative standards of behavior.” Second, any rejection by decision-makers leaves both proposer *and* receiver with a $0 payoff. This means that any rejection is a strong signal of disapproval by the decision-maker (a concern that [6] had about some of the previous treatments in the literature). Finally, our design expands the set of possible offers to include hyper-generous offers allowing us to study how decision-makers react to inequality deriving from generous intentions, something that has not been discussed in the literature. All previous reports generous offers being rejected were in field experiments with subjects either from rural old Soviet Union regions [14] or in small-scale societies in New Guinea [3]. These results always came from two-player games, and were considered anomalies by the authors. For example, [14] dismiss rejections of generous offers because of Soviet education, while [3] hypothesize that these rejections could be the result of a gift-giving culture, in which accepting large gifts establishes the receiver as a subordinate. [15] also mention a small number of generous offers being rejected.

Our main result is that neutral decision-makers have strong preferences for equality over intentions as they reject *both* selfish and generous offers to avoid inequality (from now on we will denote any offer of more than $5 as a “generous offer”). This result is robust to a series of modifications to the decision-maker’s payoffs, such as different “flat fee” payoffs or the introduction of a monetary cost to rejecting offers. Such an outcome is consistent with [16] who report that neutral third-parties (in dictator games) state that hyper-generous offers are significantly less “appropriate” than even splits. However, it is at odds with some previous results that point to intentions as one of the main drivers behind social preferences (e.g., [6]) suggesting that more research is needed in this area.

## Experimental design

As subjects arrived to the lab, they were seated randomly in front of a terminal and the initial instructions were read aloud. In these instructions we announced that:

1. The experiment had three rounds and instructions for each round would be read immediately before the beginning of each round.
2. Each subject would be assigned a player type (A, B or C) which they would keep through the experiment.
3. Each round, subjects would be randomly assigned to a different group of three players (that always included one subject of each type).
4. Only one of the rounds, randomly chosen by the computer, would be chosen for the final payoffs.
5. No feedback on other subject’s choices or payoffs would be given before the end of the session.

A time-line of the experiment is shown in Table 1.

**Table 1:** Time-line of the experiment.

Step 1	Step 2	Step 3	Step 4
Read general instructions	Read instructions for Round 1	Round 1	Read instructions for Round 2
Assign player type	Assign players to group	No feedback	Assign players to new group
Step 5	Step 6	Step 7	Step 8
Round 2	Read instructions for Round 3	Round 3	Info on results for all games
No feedback	Assign players to group	No feedback	Final payoff info

### Baseline

**Fig 1:**
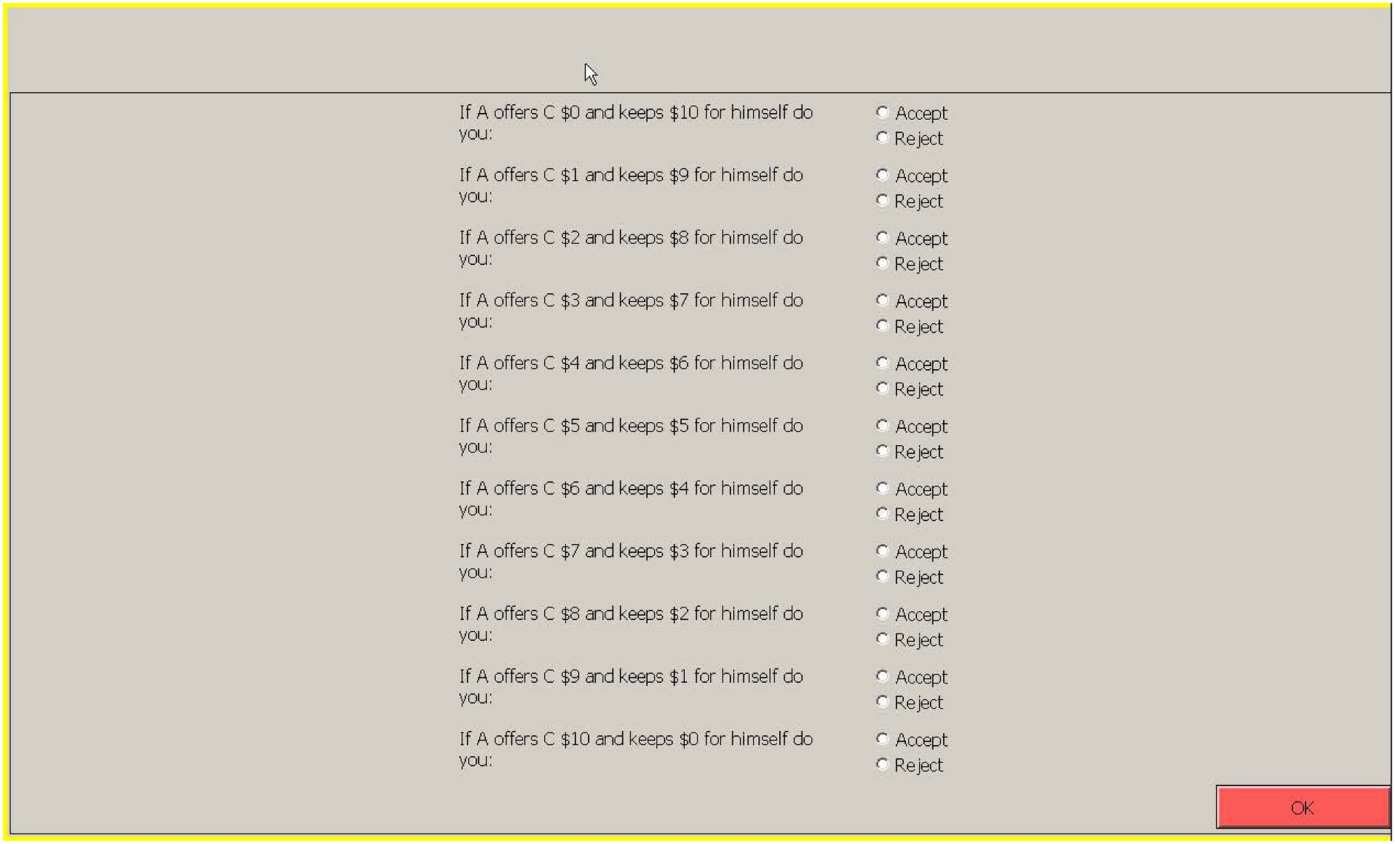
Arbiter’s screen-shot.

In the baseline design, A players are assigned the role of proposer and must make a suggestion on how to split $10 with player C who is a “passive” receiver and has no active role in the game. In the meantime (and without knowing the proposal made by the proposer) B players are assigned the role of arbiter and have to fill in a strategy profile (screen-shot in Fig 1) accepting or rejecting all potential offer that proposers could make. Arbiters get paid a “flat fee” for their decision whatever the outcome of the game, while the payoff of both proposer and receiver depend on the arbiter’s choices and the offer made by the proposer. If the offer from the proposer is accepted, the split goes as suggested, if rejected, then both proposer and receiver get $0 for the round. This payoff structure is known by all subjects when making their decisions. For arbiters we have three different “flat fee’ payoffs; a low (L) payoff of $3, a normal (N) payoff of $5, and a high (H) payoff of $12. Having these three different payoffs will allow us to test whether arbiters take into account their relative payoff when accepting or rejecting the different offers.

### Robustness check

In addition to the different flat-fee payoffs to arbiters, we introduce a “Costly Rejection” treatment as a robustness check to our results. This treatment has the same structure as the Baseline, but now arbiters are penalized with $1 if the game ends in a rejection. The objective of this penalization is to study the relative “fragility” of subjects’ aversion to selfish intentions and inequality. If the concern for selfish intentions is stronger (weaker) than that for inequality, then we should observe a relatively larger (smaller) drop in the rejection rate of generous offers than of selfish offers.

In other words, once we put a price on rejecting offers we can study the reaction of subjects to a change in prices and infer how strong is the relative aversion of subjects to selfish intentions and inequality. To make this robustness test more stringent, we only introduce it in the High or Low payoff format. So, the payoffs for the Costly Rejection robustness check are:

- Low Payoff (L-1): arbiter gets paid $3 if the proposer’s offer is accepted and $2 if rejected.
- High Payoff (H-1): arbiter gets paid $12 if the proposer’s offer is accepted and $11 if rejected.

### 2UG

Finally, in all sessions, one of the rounds will be a 2UG game. This is a regular ultimatum game but with the 3-player group structure. In it, A makes two independent suggestions on how to split $10; one to B, the other to C. As in the baseline, we use the strategy method to elicit B and C’s preferences over the offers made to them. If B (C) rejects the offer that A made *to him*, then B (C) gets $0 for the round. If, instead B (C) accepts the offer, then the split goes as suggested. A’s payoff is randomly chosen from one of the two outcomes. If the selected game turns out to be a rejection, then A gets $0 for the round, if accepted, then A gets her part of the proposal. The purpose of randomizing A’s payoffs is to prevent portfolio effects and to make payoffs fair across all subject types.

The 2UG game is introduced in our sessions for three reasons. The first one is to create, in most sessions, a “break” between our treatments of interest allowing us to recreate a “first-shot” scenario in the third round of the session. Secondly, we use the 2UG as a control, to verify whether or not our subjects understand the strategy method interface. Finally, the results of the 2UG game should help to confirm that arbiters took seriously the possibility of generous offers when filling their strategy profiles.

## Results

The experiment was run with a total of 234 undergraduates from both Universitat Pompeu Fabra (UPF) in Barcelona, and the University of California Santa Cruz (UCSC) in Santa Cruz, California (UCSC IRB approved this study as Exempt (category 2) as part of LEEPS Lab protocol HS1240 on August 26, 2008. All subjects provided written informed consent). Each session had 3 rounds and lasted on average 30 minutes. The mean earnings at UCSC were of $4.5 and at UPF of €4.35 plus a show-up fee ($5 and €3) (from now on, we will use the dollar sign to include both euros and dollars). The show-up fee was announced only at the end of the experiment, as we believe that while most subjects are aware of the rule of a “show-up fee” not announcing it until the end of the experiment adds pressure to the arbiters would their decisions result in a rejection. Subjects were recruited through the ORSEE systems of each university [17], and were required not to have any previous experience in bargaining games. Details on ordering and number of observations for each session can be found in Tables 2 and 3. Additionally, Supporting Information S1 Appendix shows that there are no significant ordering effects.

**Table 2:** Participants per treatment.

Treatment Order/Location	Barcelona	Santa Cruz
N2H	18	21
N2L	18	21
(H-1)2(L-1)	-	33
(L-1)2(H-1)	-	48
L2H	-	12
2NL	18	-
2NH	15	-
H2N	15	-
L2N	15	-

Treatment ordering and number observations for all type (A,B,C) of subjects.

**Table 3:** Arbiters by treatment and location.

	Barcelona	Santa Cruz	Total
N	33	14	47
H	16	11	27
L	16	11	27
H-1	-	27	27
L-1	-	27	27

Number of arbiter observations per treatment and location along with the total across both locations.

We begin the analysis of our data by looking at the baseline treatments in Section, and then move to the analysis of the Costly Rejection in section. The 2UG outcomes can be found in Supporting Information S2 Appendix.

### Baseline

**Fig 2:**
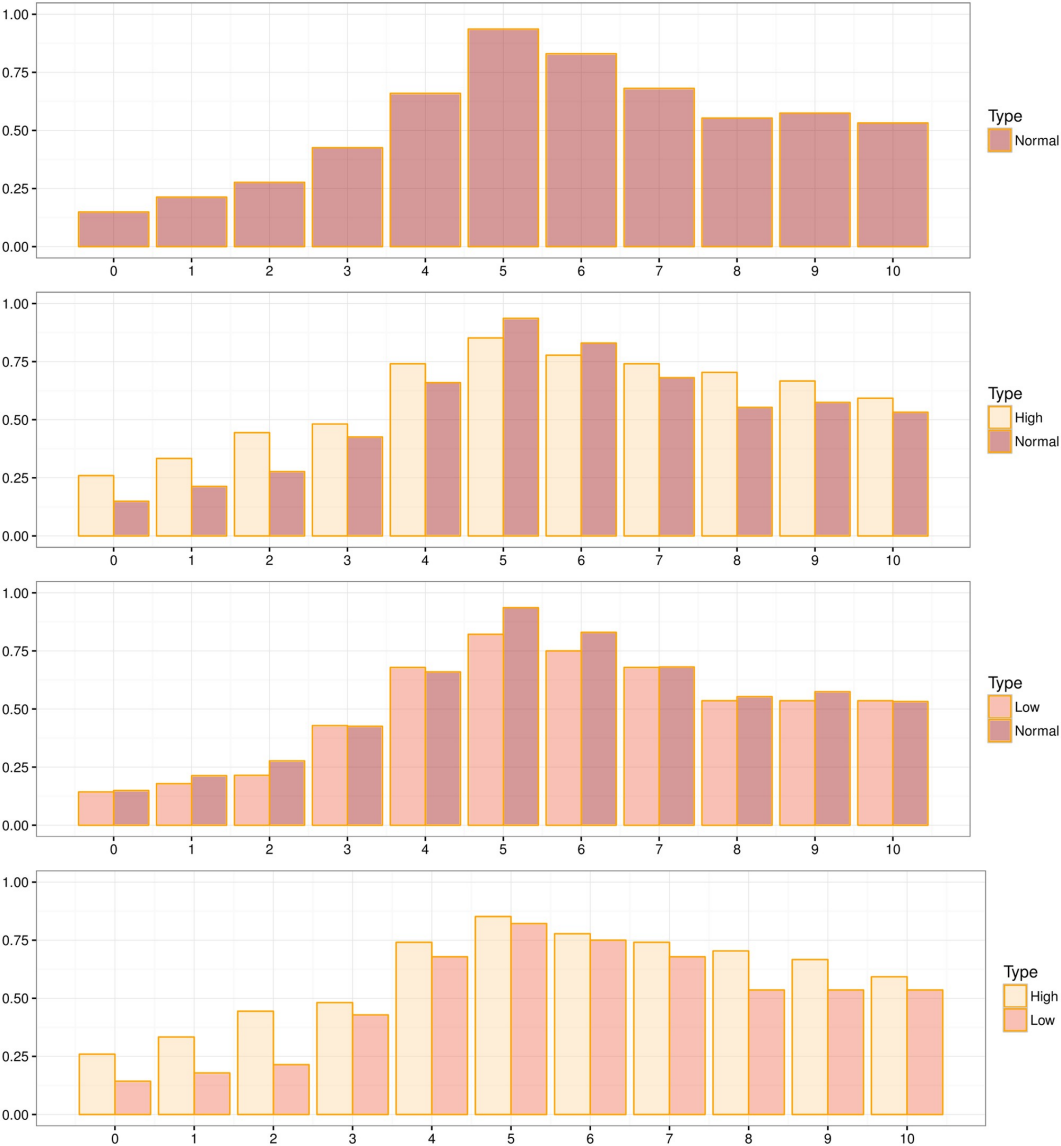
Acceptance rates for baseline treatments. For each graph, in the vertical axis we plot the percentage of acceptances, in the horizontal axis, the offer.

Fig 2 presents the percentage of acceptances for each potential offer. From top to bottom we see the acceptances rates for treatment N, the comparison between H and N, the comparison between L and N, and finally that between H and L. Two things immediately stand out: The first one is that there is a significant number of generous offers being rejected; the second one is that the results in all treatments look very much alike despite the big payoff differences of arbiters. Indeed, using a pairwise Epps-Singleton test we see no statistical differences between the distribution of acceptances in High and Low (*p*-value = 0.600), High and Normal (*p*-value = 0.579), or Low and Normal (*p*-value = 0.999). A two-sided Fisher test comparing individual acceptance rates for each potential offer confirms this result (Table 4). Notice that since we are testing a family of eleven simultaneous hypotheses, one would want to control for false positives using a Bonferroni correction shifting the significance cutoff from the conventional *p*-value of 0.05 to a “corrected” one of 0.0045. Yet, since no test is significant in Table 4 (all *p*-values are greater than 0.05), we do not need to apply the correction.

**Table 4:** Two-sided Fisher *p*-values.

	$0	$1	$2	$3	$4	$5	$6	$7	$8	$9	$10
L = H	1.000	0.775	0.596	1.000	1.000	0.141	0.550	1.000	1.000	0.810	1.000
H = N	0.355	0.280	0.202	0.808	0.604	0.250	0.759	0.792	0.226	0.469	0.636
L = H	0.329	0.227	0.089*	0.789	0.768	1.000	1.000	0.768	0.269	0.412	0.787

Furthermore, when we look at decisions at the individual level we observe that subjects do not significantly change their behavior across the different payoffs. A within subject Wilcoxon matched-pairs signed-rank test shows that the preferences of subjects are robust across the different payment level (*p*-value = 0.375, *p*-value = 0.161, and *p*-value = 0.082 when comparing N to L, N to H, and L and H respectively). This seems to indicate that arbiters pay little attention to their payoffs when making decisions, suggesting that we are close to observing the true normative preferences of subjects, free of any strategic or monetary concerns.

- **Result 1:** *In the Baseline setup we observe no statistical differences in rejection patterns across treatments*.

To check whether or not intentions play a role in the preferences of arbiters we define “absolute inequality” as the absolute value of the difference between A and C’s payoff if the offer were accepted. We then run a Probit model with the binary accept/reject outcome as dependent variable, and dummies for order (First), treatment (High, Low), location (Where), and distance to the fair split. The coding for the distance dummies includes the distance to the even split and the direction of this distance. So, for example, Dist3l is the dummy for the $2 offer (which is 3 dollars to the left of $5) and Dist2r is the dummy for an offer of $7 (which is 2 dollars to the right of $5). The results can be found in Table 5 where Column 3 has the full specification of the Probit model, and column 4 includes the interactions between treatment and distance (see Supporting Information S1 Table for the interactions). In all models the errors are clustered at the individual level.

**Table 5:** Probit model of accepted offers.

	(1) Accept	(2) Accept	(3) Accept	(4) Accept
Low	-0.0752	0.0411	0.0414	-0.455
	(0.137)	(0.170)	(0.201)	(0.306)
High	0.176	0.330	0.380	-0.299
	(0.159)	(0.213)	(0.249)	(0.396)
First		0.237	0.277	0.277
		(0.153)	(0.178)	(0.179)
Where		-0.0632	-0.0802	-0.0781
		(0.226)	(0.264)	(0.265)
Dist1l			-0.704***	-1.109***
			(0.174)	(0.280)
Dist2l			-1.346***	-1.713***
			(0.216)	(0.305)
Dist3l			-1.719***	-2.116***
			(0.237)	(0.319)
Dist4l			-1.931***	-2.324***
			(0.238)	(0.330)
Dist5l			-2.141***	-2.569***
			(0.256)	(0.349)
Dist1r			-0.367**	-0.564*
			(0.132)	(0.238)
Dist2r			-0.679***	-1.053***
			(0.174)	(0.276)
Dist3r			-0.971***	-1.390***
			(0.184)	(0.292)
Dist4r			-0.971***	-1.336***
			(0.202)	(0.290)
Dist5r			-1.072***	-1.444***
			(0.211)	(0.294)
Cons	0.0752	-0.0923	0.975***	1.330***
	(0.104)	(0.188)	(0.239)	(0.315)
*N*	1122	1122	1122	1122
Interaction	No	No	No	Yes

* *p* < 0.10,

** *p* < 0.05,

*** *p* < 0.01

The results show that the dummies for distance are not only negative and highly significant, but that their coefficients follow an (almost) perfectly monotonic pattern. The further away an offer is from $5 the lower the probability of being accepted. This result is unchanged by the inclusion of the interactions since, even if the signs for the treatment dummies change, their effect and that of the interactions continue to be statistically indistinguishable from zero.

- **Result 2:** *The greater the absolute inequality, the lower the probability of the proposal being accepted*.

However, in Fig 2 we see that the rejection patterns are not perfectly symmetric around the fair split as, for the same amount of absolute inequality, generous offers are more likely to be accepted than selfish ones. The difference is significant (see *p*-values of Two-sided Fisher test in Table 6) and points towards an effect of selfish intentions on arbiters. As in Table 4, one would want to correct for multiple hypothesis testing. In this case, our family consists of five tests, so the significance threshold shifts from the conventional *p*-value of 0.05 to 0.01 when applying the Bonferroni correction. Such a correction would eliminate any statistically significant results for treatments H and L in Table 6, while showing significant results in the N treatment for the most extreme cases.

**Table 6:** Two-sided Fisher test *p*-values.

Treatment	$4 = $6	$3 = $7	$2 = $8	$1 = $9	$0 = $10
L	0.768	0.106	0.026	0.011	0.004
H	1.000	0.093	0.098	0.029	0.027
N	0.048	0.011	0.006	0.001	<0.001

- **Result 3:** *In the baseline treatments, arbiters are less willing to tolerate inequality when it is the result of a selfish offer*.

### Costly-rejection

**Fig 3:**
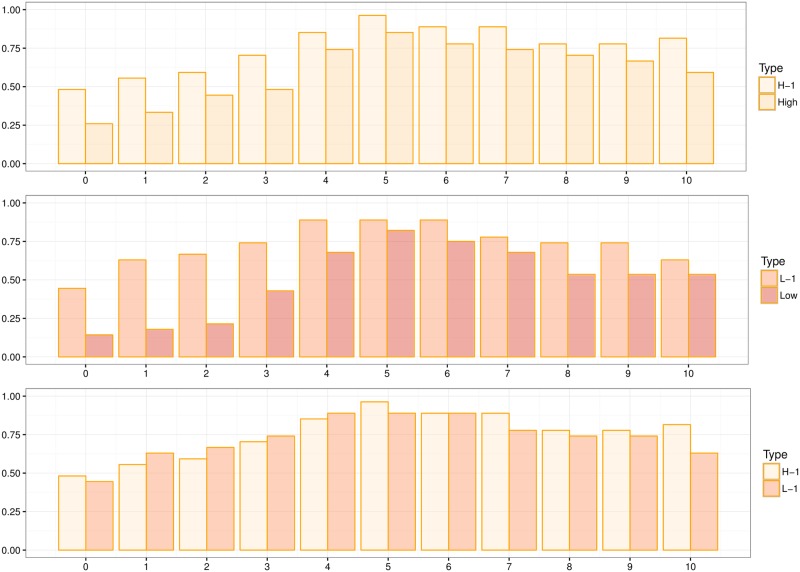
Acceptance rates of L-1 and H-1 plotted against their baseline counterparts and a comparison of L-1 and H-1. For each graph, in the vertical axis we plot the percentage of acceptances, and in the horizontal axis the offer.

In Fig 3 we present the results of the costly-rejection treatments and compare them to their baseline counterparts; H-1 and H in the top panel, and L-1 and L in the middle, and H-1 and L-1 at the bottom.

As in the Baseline treatments, *both* selfish and generous offers continue to be rejected in all costly treatments. But more surprising is that H-1 and L-1 look almost identical. A Two-sided Fisher test finds no differences across individual offer acceptance rates (see Supporting Information S2 Table), nor does an Epps-Singleton test find any differences when comparing the whole distribution (*p*-value = 0.907). An identical result happens when we use only first round data points (*p*-value = 0.969). In this case we have 11 subjects playing H-1 in the first round and 16 subjects playing L-1. Moreover, when comparing the acceptances within subject using a Wilcoxon matched pairs sign-rank test we see no differences at the subject level (*p*-value = 0.617). So, even when the relative costs of rejecting offers are wide apart, arbiters behave in a similar manner under both costly treatments. This confirms Result 1; our game structure allows us to observe the normative standards of behavior of arbiters.

- **Result 4:** *Even with widely different relative rejection costs, there is no significant difference across treatments in the Costly-Rejection setup*.

On the other hand, we do see some differences when comparing the Costly Rejection treatments and their Baseline counterparts. These differences arise mostly in the left of the distributions; that is, where the selfish offers are. As can be seen, once we introduce a cost to rejecting offer, arbiters are less willing to punish selfish behavior (especially in the L-1 treatment), but continue to reject generous offers at the same rate than in the Baseline treatment (see Table 7 for the *p*-value of one-sided Fisher tests comparing treatments). Given that in Table 7 we have a family with 11 tests, using the Bonferroni correction to test for multiple hypothesis would eliminate all differences across comparisons as the new significance threshold would shift to 0.0045. Yet, one should interpret this correction with care since, given the pattern of the *p*-values in Table 7 and the patterns in Fig 3, we suspect that this might be one case in which the Bonferroni correction is too stringent.

**Table 7:** One-sided Fisher *p*-values comparing total acceptances per treatment.

	$0	$1	$2	$3	$4	$5	$6	$7	$8	$9	$10
L = L-1	0.01	0.01	0.01	0.01	0.05	0.37	0.16	0.30	0.09	0.09	0.33
H = H-1	0.07	0.08	0.20	0.08	0.25	0.17	0.23	0.14	0.37	0.27	0.06

It appears that introducing a cost to rejecting offers reduces the concerns of arbiters for “intentions,” while maintaining their concerns for inequality. This indicates the relative fragility that concerns for selfish intentions have relative to concerns for inequality. A two-sided Fisher-test confirms the symmetry in the distributions of acceptance rates in both costly treatments (see Table 8). In this case we have a family of five tests, so the cutoff for significance would shift to a *p*-value = 0.01, making all comparisons of Table 8 statistically indistinguishable.

**Table 8:** Two-sided Fisher *p*-values.

	$4 = $6	$3 = $7	$2 = $8	$1 = $9	$0 = $10
L-1	1.000	1.000	0.766	0.559	0.275
H-1	1.000	0.175	0.241	0.148	0.021

- **Result 5:** *The introduction of a cost to rejecting offers reduces the influence of intentions in the acceptance pattern of arbiters, pointing towards the payoff inequality between proposer and receiver as the main reason for rejecting an offer*.

Finally, to offer an overall picture of the whole experiment and its different treatments, Table 9 presents a Probit model comparing N to the High and Low payoff treatments for both Baseline and Costly Rejection. The results show that treatments are not really significant, but that, in all cases, the distance from the fair split is highly significant, and the probability of acceptance decreases as absolute inequality increases. Both models include the interactions between the different treatments and the absolute distance (see Supporting Information S3 Table for details). Only the interaction for the selfish offers in the case of the L-1 treatment are mildly significant and positive, lending support to the idea that the aversion for selfish intentions flounders once rejection costs are high.

**Table 9:** Probit model comparing each treatment to baseline N treatment.

	(1)	(2)
	Baseline	Costly
First	0.277	0.0805
	(0.179)	(0.191)
Where	-0.0781	-0.0495
	(0.265)	(0.293)
Low	-0.455	
	(0.306)	
High	-0.299	
	(0.396)	
L-1		0.333
		(0.594)
H-1		-0.249
		(0.487)
Dist1r	-0.564*	-0.568*
	(0.238)	(0.236)
Dist2r	-1.053***	-1.054***
	(0.276)	(0.272)
Dist3r	-1.390***	-1.390***
	(0.292)	(0.287)
Dist4r	-1.336***	-1.336***
	(0.290)	(0.285)
Dist5r	-1.444***	-1.444***
	(0.294)	(0.290)
Dist1l	-1.109***	-1.112***
	(0.280)	(0.275)
Dist2l	-1.713***	-1.712***
	(0.305)	(0.300)
Dist3l	-2.116***	-2.117***
	(0.319)	(0.314)
Dist4l	-2.324***	-2.321***
	(0.330)	(0.325)
Dist5l	-2.569***	-2.564***
	(0.349)	(0.344)
Constant	1.330***	1.475***
	(0.315)	(0.290)
*N*	1122	1111
Interaction	Yes	Yes

Standard errors in parentheses

* *p* < 0.10,

** *p* < 0.05,

*** *p* < 0.01

## Conclusion

From conflict resolution to evolutionary biology, social preferences are an object of study in many different research areas. A complete understanding of how they operate should be a priority, yet the results we have on the relative importance of selfish intentions versus inequality aversion appear inconclusive; while [6, 8] claim that intentions are the main driver behind the actions of third parties, [12] show that subjects respond more to outcomes than to intentions.

Our contribution to this literature consists in designing a game to observe the truly normative preferences of neutral third-parties. By paying arbiters a fixed amount whatever their decision we can observe the social preferences of subjects free of any strategic or monetary concerns.

The results show that arbiters are willing to reject a significant amount of selfish and generous offers in order to reduce the inequality of payoffs between participants. This is surprising, since rejecting an offer implies leaving both proposer and receiver with a payoff of $0. We interpret this result as a preference of arbiters for equality over the (selfish) intentions of the proposer. The introduction of a cost for rejecting an offer seems to confirm our results as the aversion of arbiters to the (selfish) intentions of proposers seems to disappear in this case, while the aversion of arbiters to inequality remains. A secondary result of our experiment is that arbiters are not efficiency seekers, as they are willing to leave all subjects with a payoff of $0 rather than accepting an uneven offer, even if this uneven offer is a generous one.

We believe our results are evidence that inequality aversion plays a larger role in social preferences than that suggested by the previous literature.

## Supporting information

S1 Appendix Ordering effects. (PDF)

S2 Appendix 2UG results. (PDF)

S3 Appendix Instructions for subjects. (PDF)

S1 Table Interaction terms Table 4. (PDF)

S2 Table Two-sided Fisher tests. (PDF)

S3 Table Interaction terms Table 8. (PDF)

S1 Data Data of the experimental sessions. (ZIP)
