# Supplementary material for: Equality over intentionality: The normative social preferences of neutral third-parties
Source: PLoS One. 2018 Nov 14;13(11):e0205240. doi: 10.1371/journal.pone.0205240 (PMC6235270; doi:10.1371/journal.pone.0205240)

Appendix:

A 2UG Results

We summarize all of B subject's observations in Figure 4. In it we present the percentage of arbiters accepting each potential offer from the proposer to the receiver (e.g. almost 60% of arbiters accept a hypothetical offer of $3 while only 30% accept one of 1). The acceptance results are slightly higher than those reported in the literature (see Camerer and Thaler (1995)), but still within the range of what would be expected. The average offer was of $3.59, which is also what would be expected in an experiment like this. These results validate both our subject pool and the software interface, but most importantly, they show that arbiters act consistently when deciding about hyper-generous offers (i.e., subjects do not randomize or "experiment" within this range of offers).Three subjects that rejected offers of $8 or more yet accepted all smaller offers. We believe that these subjects misunderstood the interface and were trying to reject offers smaller than $2. We take this as an indication that arbiters take seriously the possibility of a generous offer.

Figure 4: Acceptances of 2UG

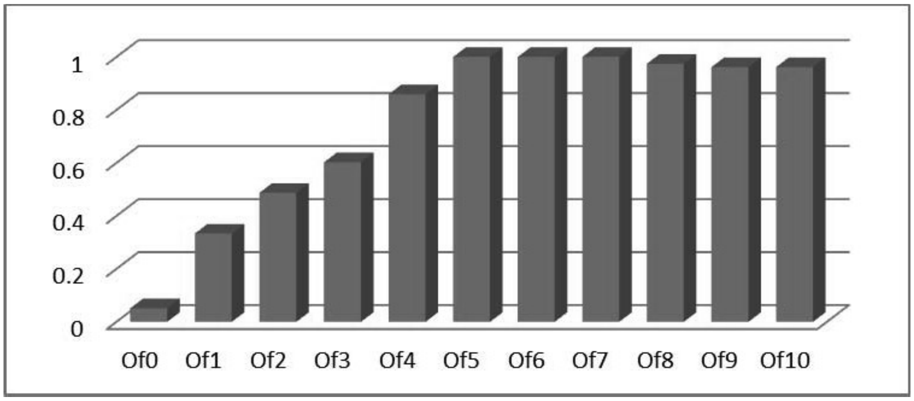

Supplement: S1 Appendix — (PDF) [file pone.0205240.s001.pdf]
