# Supplementary material for: Equality over intentionality: The normative social preferences of neutral third-parties
Source: PLoS One. 2018 Nov 14;13(11):e0205240. doi: 10.1371/journal.pone.0205240 (PMC6235270; doi:10.1371/journal.pone.0205240)

B Ordering Effects

Due to a miscommunication between the Barcelona and Santa Cruz labs we have a very unbalanced amount of first round H treatment (5) compared with third round H treatment (22). This unfortunately pollutes the ordering effects for the H treatments as a 2 tailed Fisher Test comparing first round treatments against other rounds in the experiment shows.

Table 9: Two-Sided Fisher P-values Comparing First Round Treatments to all Other Treatments

	\$0	\$1	\$2	\$3	\$4	\$5	\$6	\$7	\$8	\$9	\$10
N	0.752	0.890	0.344	0.671	0.174	1.000	0.767	0.492	0.357	0.923	0.628
H-1	0.704	1.000	1.000	0.090	0.621	1.000	1.000	1.000	1.000	1.000	1.000
H	0.091	0.030	0.010	0.165	0.238	1.000	1.000	1.000	1.000	0.136	0.060
L	0.574	1.000	0.352	0.687	0.407	1.000	1.000	1.000	0.435	1.000	0.435
L-1	1.000	0.448	0.692	1.000	0.056	0.549	0.549	1.000	0.662	0.662	0.448

While most treatments have no ordering effects, the LHT of the H treatment seems to be significantly affected by ordering. If we look at Figure 5, we can see that while last round pattern of acceptances does look like those in the rest of treatments, first round H acceptances looks pretty random. As mentioned, we believe that this is due to the low number of observations of H in the first round, and that if we had more observations we would see no ordering effects.

Figure 5: Acceptance Rates for H for First (n=5) and Third (n=22) Round

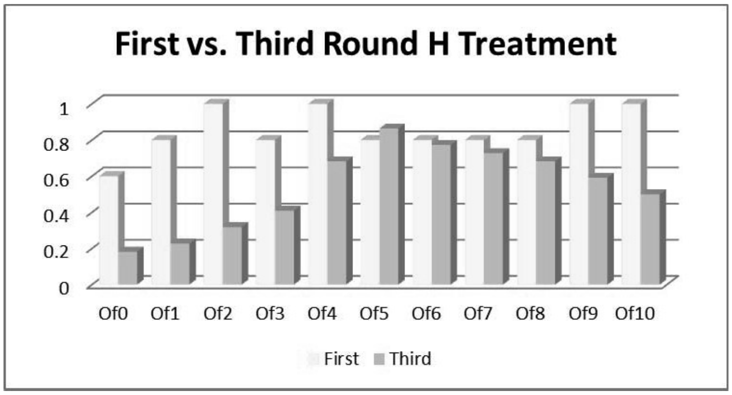

Supplement: S2 Appendix — (PDF) [file pone.0205240.s002.pdf]
