# Supplementary material for: Equality over intentionality: The normative social preferences of neutral third-parties
Source: PLoS One. 2018 Nov 14;13(11):e0205240. doi: 10.1371/journal.pone.0205240 (PMC6235270; doi:10.1371/journal.pone.0205240)

E Instructions L2H

Welcome! This is an economics experiment. You will be a player in many periods of an interactive decision-making game. If you pay close attention to these instructions, you can earn a significant sum of money. It will be paid to you in cash at the end of the last period. It is important that you remain silent and do not look at other people's work. If you have any questions, or need assistance of any kind, please raise your hand and we will come to you. If you talk, laugh, exclaim out loud, etc., you will be asked to leave and you will not be paid. We expect and appreciate your cooperation today.

This experiment has three different rounds. Before each round the specific rules and how you will earn money will be explained to you. In each round there will always be three types of players: A, B and C. You will be assigned to a type in Round 1 and will remain this type across all three rounds. Only one of the three rounds will be used for the final payoffs. This round is chosen randomly by the computer. The outcomes of each round are not made public until the end of the session (i.e. after round 3). Each round the groups are scrambled so you will never make offers or decide for the same player in two different rounds.

Round 1:

The first thing that you will see on your screen is your player type.

You will then be assigned to a group consisting of three players: an A type, B type and C type.

Player A will be endowed with $10 which he will split with player C. In order to do so Player A will have to input the amount he is willing to offer Player C. Player A will only be able to make integer offers (full dollars), so A will not be able to break its offer into cents.

While player A is deciding how much to offer player C, player B will be filling out a binding "strategy profile". The strategy profile has an "accept or reject" button for each potential offer from A to C (from $0 to $10). Player B's binding decision to accept or reject A's offers to C will be done before he knows the actual offer made by A.

A's decision: How to split an endowment of $10 with Player C by making him an offer between $0 and $10. If the offer is of $X, A will be keeping for himself 10-X.

B's decision: Before knowing the offer from A to C, B will fill a binding "strategy profile" deciding whether he accepts or rejects every potential offer from A to C. This decision is made without knowing the offer from A to C.

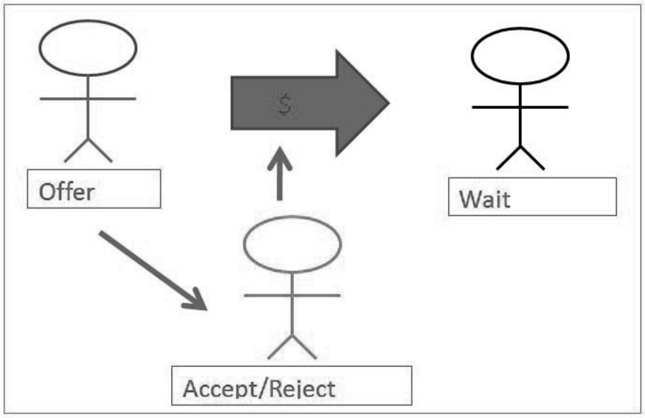

It is very important for A to realize that he is going to write the amount he wants to offer C and not how much he wants to keep.

Payoff for Round 1:

If B accepts the offer from A to C, then they split the $10 as suggested by A.

If B rejects the offer from A to C, then both (A and C) get $0.

B will get paid $3 no matter what is the outcome.

Timing and Payoffs:

1. B fills a strategy profile with all potential offers from A to C.

2. A decides how much to offer C (say X)

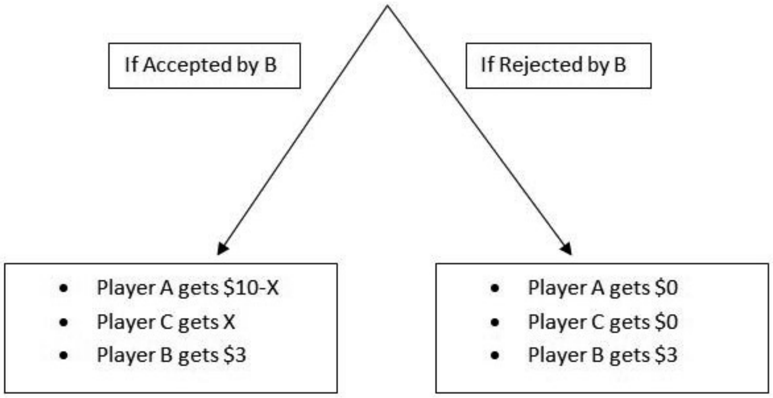

<u>Round 2</u>:

As mentioned at the beginning of the experiment you will keep your player type across the whole session. So A players are still A, B are B and C are C.

In this round type A players will be endowed with $20 and will have to make TWO offers:

1. How to split $10 with player B.

2. How to split $10 with player C.

As in Round 1 a binding "strategy profile" will be filled by B and C players before they know the offer made to *them*.

It is very important to notice that B and C players are making decisions concerning their own payoffs.

<u>A's decision</u>: How to split $10 with B and how to split $10 with A.

Each offer is independent. So the outcome of the offer to B has no effect on the outcome of the offer to C.

Payoffs for A will be as in Round 1 (if he offers X and the offer is accepted he gets $10-X, if the offer is rejected both him and the rejecting player get 0).

B and C players will get paid X or 0 depending if the accepted or rejected the offer made directly to them.

In order to make payoffs equitable for this round, A's payoff for this round will be chosen at random between one of the two outcomes (offer to B and offer to C). B and C's decision: Before knowing the offer made to them by Player A, B and C will fill a binding "strategy profile" deciding if they accept or reject *every potential offer made directly to them.*

If the offer from A is accepted, then the split is done as proposed by A. If the offer is rejected both the receiver and A get $0 as the outcome for this round.

<u>Timing and Payoff for Round 2</u>:

1. Each receiver fills a strategy profile with all potential offers that A could make them.

2. A decides how much to offer C and B (say X)

3. Payoffs for B and C will be the outcome of their particular game with A.

4. To make outcomes equitable, the computer will choose randomly one of the two outcomes to be A's payoff for the round.

For each offer made from A to the other members of his group:

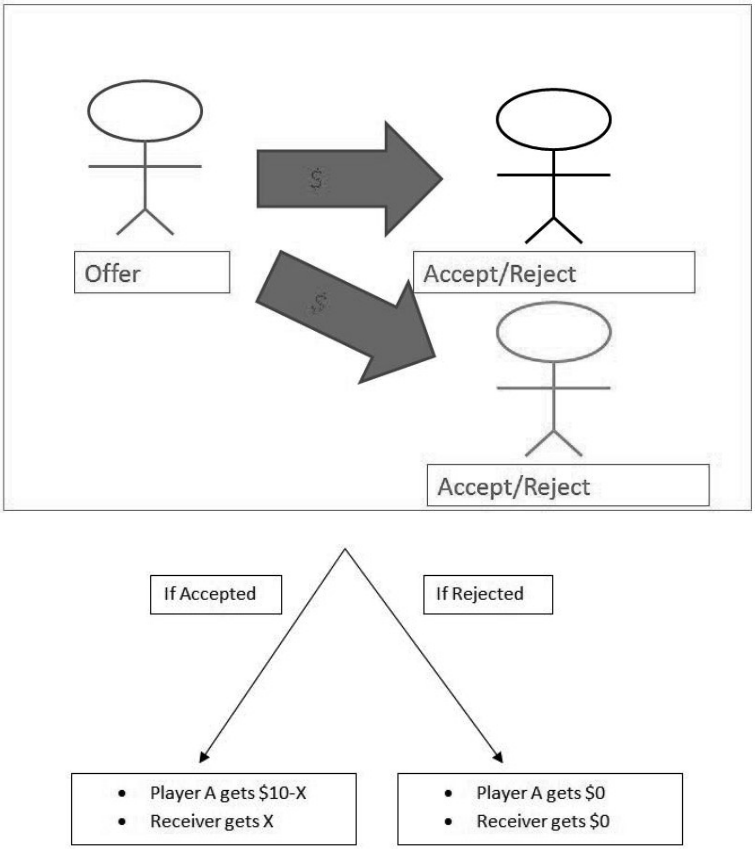

<u>**Round 3**</u>:

As mentioned at the beginning of the experiment you will remain your player type across the whole session.

This round is very similar to round 1. You will now be re-scrambled into groups of three subjects (one A, one B and one C subject).

A will be endowed with $10 and must decide how to split them with C.

B's role is exactly the same as that in round 1: Before knowing the offer from A to C, B will fill a "strategy profile" deciding whether he accepts or rejects *every potential offer from A to C*.

If the offer from A to C is accepted by B, then the split is done as proposed by A. If B rejects the offer, then both A and C receive $0 for this round.

B's payoff in this round is a flat $12 fee, whatever his decision and outcome of the round. So, the only change between Round 1 and Round 3 is that player B, is getting paid a different amount.

<u>Timing and Payoffs</u>:

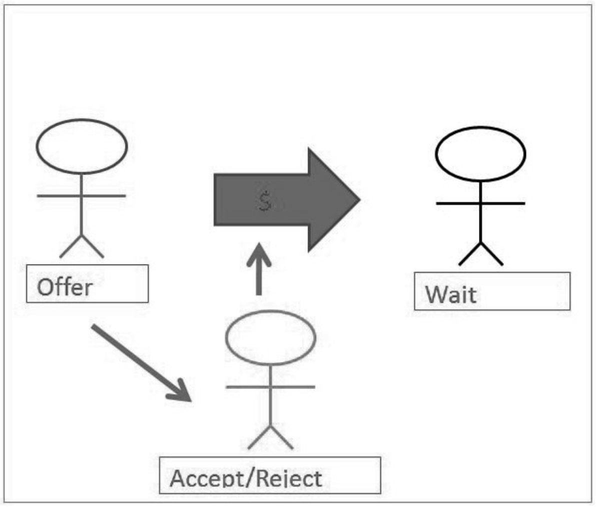

1. B fills a strategy profile with all potential offers from A to B.

2. A decides how much to offer C (say X)

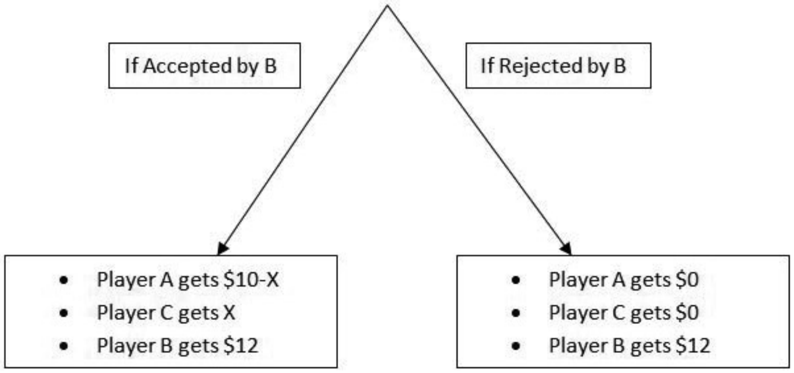

Supplement: S3 Appendix — (PDF) [file pone.0205240.s003.pdf]
