# Supplementary material for: Equality over intentionality: The normative social preferences of neutral third-parties
Source: PLoS One. 2018 Nov 14;13(11):e0205240. doi: 10.1371/journal.pone.0205240 (PMC6235270; doi:10.1371/journal.pone.0205240)
Supplement: S1 Table — (PDF) [file pone.0205240.s004.pdf]

## C Interactions

| | (1) Accept | (2) Accept | (3) Accept | (4) Accept |
|---|---|---|---|---|
| Dist1l*Low | | | | 0.134 |
| | | | | (0.0840) |
| Dist2l*Low | | | | 0.118 |
| | | | | (0.0904) |
| Dist3l*Low | | | | 0.0524 |
| | | | | (0.101) |
| Dist4l*Low | | | | 0.0805 |
| | | | | (0.110) |
| Dist5l*Low | | | | 0.109 |
| | | | | (0.109) |
| Dist1r*Low | | | | 0.0350 |
| | | | | (0.0687) |
| Dist2r*Low | | | | 0.112 |
| | | | | (0.0814) |
| Dist3r*Low | | | | 0.0973 |
| | | | | (0.102) |
| Dist4r*Low | | | | 0.0760 |
| | | | | (0.109) |
| Dist5r*Low | | | | 0.119 |
| | | | | (0.103) |
| Dist1l*High | | | | 0.165 |
| | | | | (0.0945) |
| Dist2l*High | | | | 0.140 |
| | | | | (0.0959) |
| Dist3l*High | | | | 0.252* |
| | | | | (0.0995) |
| Dist4l*High | | | | 0.205* |
| | | | | (0.0967) |
| Dist5l*High | | | | 0.195* |
| | | | | (0.0881) |
| Dist1r*High | | | | 0.0323 |
| | | | | (0.0597) |
| Dist2r*High | | | | 0.144* |
| | | | | (0.0679) |
| Dist3r*High | | | | 0.235** |
| | | | | (0.0800) |
| Dist4r*High | | | | 0.177 |
| | | | | (0.0973) |
| Dist5r*High | | | | 0.145 |
| | | | | (0.106) |
| $N$ | 1122 | 1122 | 1122 | 1122 |

* $p < 0.10$, ** $p < 0.05$, *** $p < 0.01$

Table 10: Interactions of Table 4
