# Supplementary material for: Equality over intentionality: The normative social preferences of neutral third-parties
Source: PLoS One. 2018 Nov 14;13(11):e0205240. doi: 10.1371/journal.pone.0205240 (PMC6235270; doi:10.1371/journal.pone.0205240)
Supplement: S2 Table — (PDF) [file pone.0205240.s005.pdf]

D Two Sided Fisher Tests

Two-Sided Fisher test comparing treatments for the Costly Rejection setup.

	\$0	\$1	\$2	\$3	\$4	\$5	\$6	\$7	\$8	\$9	\$10
L-1 = H-1	1.000	0.782	0.779	1.000	1.000	0.610	1.000	0.467	1.000	1.000	0.224

Table 12: Two-Sided Fisher p-values
