# Supplementary material for: Equality over intentionality: The normative social preferences of neutral third-parties
Source: PLoS One. 2018 Nov 14;13(11):e0205240. doi: 10.1371/journal.pone.0205240 (PMC6235270; doi:10.1371/journal.pone.0205240)
Supplement: S3 Table — (PDF) [file pone.0205240.s006.pdf]

Table 11: Interactions for Table 8

(a) Baseline

Dist1r*Low	0.312
	(0.278)
Dist2r*Low	0.586*
	(0.290)
Dist3r*Low	0.549
	(0.328)
Dist4r*Low	0.494
	(0.356)
Dist5r*Low	0.602
	(0.342)
Dist1l*Low	0.637*
	(0.294)
Dist2l*Low	0.600*
	(0.300)
Dist3l*Low	0.382
	(0.370)
Dist4l*Low	0.465
	(0.433)
Dist5l*Low	0.559
	(0.470)
Dist1r*High	0.286
	(0.260)
Dist2r*High	0.655*
	(0.282)
Dist3r*High	0.881**
	(0.318)
Dist4r*High	0.726
	(0.372)
Dist5r*High	0.635
	(0.386)
Dist1l*High	0.718
	(0.375)
Dist2l*Hight	0.617
	(0.346)
Dist3l*High	0.925*
	(0.380)
Dist4l*High	0.837*
	(0.389)
Dist5l*High	0.865*
	(0.385)
N	1122

(b) Costly

dist1r*H-1	-0.001
	(0.446)
dist2r*H-1	0.484
	(0.631)
dist3r*H-1	0.364
	(0.619)
dist4r*H-1	0.310
	(0.618)
dist5r*H-1	0.549
	(0.623)
dist1l*H-1	0.367
	(0.489)
dist2l*H-1	0.458
	(0.537)
dist3l*H-1	0.559
	(0.556)
dist4l*H-1	0.669
	(0.565)
dist5l*H-1	0.726
	(0.582)
dist1r*L-1	0.568
	(0.368)
dist2r*L-1	0.593
	(0.428)
dist3r*L-1	0.810
	(0.445)
dist4r*L-1	0.756
	(0.389)
dist5r*L-1	0.550
	(0.416)
dist1l*L-1	1.116**
	(0.399)
dist2l*L-1	1.133*
	(0.453)
dist3l*L-1	1.322**
	(0.471)
dist4l*L-1	1.427**
	(0.484)
dist5l*L-1	1.200**
	(0.517)
N	1111
